# The Complex Puzzle of Interactions Among Functional Food, Gut Microbiota, and Colorectal Cancer

**DOI:** 10.3389/fonc.2018.00325

**Published:** 2018-09-05

**Authors:** Lígia A. B. M. Mendonça, Rosângela dos Santos Ferreira, Rita de Cássia Avellaneda Guimarães, Alinne P. de Castro, Octávio L. Franco, Rosemary Matias, Cristiano M. E. Carvalho

**Affiliations:** ^1^S-Inova Biotech Post Graduate Program in Biotechnology, Catholic University Dom Bosco, Campo Grande, Brazil; ^2^Post Graduate Program in Health and Development in the Central-West Region of Brazil, Federal University of Mato Grosso do Sul, Campo Grande, Brazil; ^3^Center of Proteomic and Biochemical Analysis, Post Graduate Program in Genomic Sciences and Biotechnology, Catholic University of Brasilia, Brasilia, Brazil; ^4^Post Graduate Program in Environmental Sciences and Agricultural Sustainability, Catholic University Dom Bosco, Campo Grande, Brazil; ^5^Post Graduate Program in Environment and Regional Development, University Anhanguera Uniderp, Campo Grande, Brazil

**Keywords:** functional foods, intestinal neoplasm, gut microbiota, bioactive compounds, dysbacteriosis

## Abstract

Colorectal cancer exerts a strong influence on the epidemiological panorama worldwide, and it is directly correlated to etiologic factors that are substantiated by genetic and environmental elements. This complex mixture of factors also has a relationship involving the structural dependence and composition of the gut microbiome, leading to a dysbacteriosis process that may evolve to serious modifications in the intestinal lining, eventually causing the development of a neoplasm. The gastrointestinal tract presents defense strategies and immunological properties that interfere in intestinal permeability, inhibiting the bacterial translocation, thus maintaining the integrity of intestinal homeostasis. The modulation of the intestinal microbiome and the extinction of risk factors associated with intestinal balance losses, especially of environmental factors, make cell and defense alterations impossible. This modulation may be conducted by means of functional foods in the diet, especially soluble fibers, polyunsaturated fatty acids, antioxidants and prebiotics that signal immunomodulatory effects in the intestinal microbiota, with preventive and therapeutic action for colorectal cancer. In summary, this review focuses on the importance of dietary modulation of the intestinal microbiota as an instrument for dysbacteriosis and, consequently, for the prevention of colorectal cancer, suggesting anticarcinogenic, and antiangiogenic properties. Among the intestinal modulating agents considered here are functional foods, especially flaxseed, oat and soy, composing a Bioactive Food Compound.

## Introduction

Colorectal carcinogenesis (CRC) is a neoplastic modality with a wide and varied incidence and geographical distribution (1). CRC dissemination can be observed from the alarming epidemiological panorama, and future estimates demonstrate a considerable increase in the number of new cases of and deaths from CRC (2).

CRC etiology could be based on numerous genetic and environmental changes. Damage to the intestinal environment and mutations can culminate in the development of inflammatory bowel diseases (IBDs). The IBDs are one of the major genetic risk factors and are strongly linked to changes in the composition of the gut microbiota and the intestinal cell microenvironment (3). The environmental factors involved in CRC etiology derived from deleterious effects on gut microbiota (bacterial translocation, increased intestinal permeability and the inflammatory process) are influenced by inappropriate eating habits (4), such as excessive consumption of fast (5) and fried foods, excessive salt, saturated fat, red meat, and sugary beverages, and low fiber intake (6).

Therefore, functional foods and their bioactive substances provide a viable and accessible alternative in minimizing damaged to the intestinal microenvironment. Fibers and polyunsaturated fatty acids (PUFAs), especially those of the n-3 series, are examples of functional foods that act on gut microbiota composition, decreasing the number of harmful bacteria, such as *Helicobacter pylori* (7–12). These foods also act in minimizing the inflammatory intestine process, preventing tumorigenesis promoted by CRC (13).

These etiological findings connect directly to immune system activities that show numerous and important protective strategies, such as the synthesis and secretion of mucus from goblet cells (14). This mucus released into the intestinal region is constituted by antibacterial factors and immunomodulatory molecules (15).

The gastrointestinal tract has its organization based on the presence of pattern recognition receptors (PRRs) that recognize particularities and characteristics of pathogens and the toll-like receptors that are related to the cascade of inflammatory signaling (9, 16). Therefore, it is essential to maintain the intestinal microbiome homeostasis, from the precise relationship among the quantity and quality of the resident microbial agents. Such a relationship seems to be fundamental for the maintenance of intestinal epithelial cell integrity and for adequate immune response, avoiding the development of dysbacteriotic processes (17). However, the interindividual variability, in face of endogenous and exogenous factors, determine the gut microbiota formation playing an important role in dysbacteriosis, leading or not to diseases development such as CRC (18). Birth types seems to be one of main factor that may influence in gut microbiota formation, being that, the composition of gut bacterial community is different in infants delivered by cesarean section in comparison to infants born by vaginal delivery (19, 20). Besides that, infant's lifestyle also influences the microbiota gut formation (21). In this context, the ethnicity, geography and socioeconomic variation seems to strongly influence the occurrence of a particular microorganism or not (22–24). The host genetics also could be an important influence factor of gut microbiota, as well as, xenobiotic exposure (pesticides, environmental pollution and chemical substances, improper eating habits and use of medicament). In the case of xenobiotic exposure, the authors mention that microorganisms that compound the gut microbiota can alter the chemical structure of poluents and minimize the harmful effects on host physiology, being a reliable gut microbiota for human health (25). Thus, this scenario shows that research in this line is challenging and yet to be explored and further investigated (18). Another challenge of gut microbiota study is to move beyond classification of the bacteria agents, but also to elucidate the mechanisms underlying their influence on host health (25).

In summary, this review aims to describe the dysbacteriotic process and its consequences in the composition and conditions of the gastrointestinal tract. It also aims to describe how the immune system and its agents are involved in the repair of dysbiosis and the development of CRC and how the modulation of the gut microbiota takes place in response to functional foods and their bioactive constituents.

## Colorectal carcinogenesis

Cancer is a multifaceted disease that presents abnormal and disordered cell growth as the main pathological characteristic (26). Another important feature of cancer is the alarming epidemiological framework that indicates a substantial increase in the number of deaths (13 million) and new cases (21.7 million) by the year 2030 (2).The IARC (2) and Cancer Research UK (27) emphasize that the epidemiological context of cancer by the year 2030 may be even greater, reaching 23.6 million new cases. This increase could be strongly correlated to risk factors including smoking, alcoholism, and a poor junk food diet (5) normally composed of saturated and trans fat, salt and sugars, constituting low nutritional quality (28). Other risk factors are also cited, including the high consumption of red meat and sugary drinks and a low fiber intake and sedentary lifestyle (6). In this scenario, CRC is highlighted because it is a silent disease, especially in the early stages, not presenting clear symptoms, thus substantially impairing the diagnostic process, treatment and cure. In advanced cases, signs and symptoms can be perceived, related to changes in the gastrointestinal tract that including: diarrhea or constipation, incomplete intestinal emptying, hemorrhoids, and occult blood in the eliminations, besides associated symptoms such as cramps and stomach pain, fatigue, weight loss and low red blood cell count (29–31).

Such symptoms, if diagnosed and identified early, contribute to a brief and assertive treatment, which includes local and systemic treatment. The former is characterized by a surgical process, radiation therapy and ablation, while the latter is targeted chemotherapy (29, 30, 32). CRC presents high frequency (1, 30, 33). For Arnold et al. (34), the number of new cases of this neoplasm will increase by 60% by the year 2030, representing 2.2 million affected and 1.1 million deaths. This type of cancer is reported as the third most common form in males and the second in females (26, 35), and it is considered the second cause of death worldwide. CRC ranks first in incidence and is responsible for 47,100 new cases per year, with an average mortality of 28.2/100,000 inhabitants in the countries of the West, South, North and West Central Europe (36, 37). In these countries, over the last 40 years, CRC has presented different profiles for the level of mortality related to gender, age group, advancement of health conditions, greater access to screening methods, with consequent early detection of the disease and specialized care (38).

CRC in Asian countries occupies first place in terms of mortality and incidence (39, 40). The disease has grown significantly in recent years, although to a lesser extent than in western countries (40), and it presents wide geographic variation, especially in developed countries, such as Japan, South Korea, Singapore and Malaysia (41, 42). The epidemiological history of CRC in the USA presents similar characteristics to the global picture regarding incidence and mortality. For the year 2017, 95.520 new cases of colon cancer and 93.910 new cases of rectal cancer were estimated, distributed evenly among men and women. A similar framework can be observed in relation to the number of deaths, with 27.150 among men and 23.110 among women (43). The men CRC incidence may occur due to risk factors exposure such as smoking and alcohol, for example (43, 44). In women older than 65 years, a lower CRC survival rate predominates (45). Such information, according to some authors, may be associated with oestrogens that plays an essential role in modulating the developing CRC risk. This is due to a differential gut hormone receptors expression, although this role has not being well elucidated (46, 47). In Brazil, CRC presents heterogeneity, distributed unevenly among regions. In 2018, 17.380 new cases were estimated among men and 18.980 among women, due to the differences associated with biological and pathophysiological factors (48), like age, since women have a longer life expectancy, a condition that makes them more vulnerable due to hormonal changes, a condition similar to the global framework (43, 46, 47). In addition to gender, there are other etiologic factors that may be related to CRC, including advanced age, genetic alterations, erroneous eating habits, sedentary lifestyle, obesity, alcoholism, smoking, contact with microorganisms and diseases such as: ulcerative colitis (UK) (49), Crohn's disease (CD), familial polyposis (FP), and Lynch syndrome (LS) (35). These factors, if not detected and corrected in advance, guarantee the development of initial lesions in the lining of the colon. These lesions are also commons in IBDs that are secondary CRC causes of and are characterized by long-term intestinal inflammation (50) and genetic alteration, culminated in oxidative stress, cellular proliferation, and activation of immune system (51).

These lesions, if not treated, can evolve in the deeper muscular layers and throughout the intestinal wall, culminating in the development of CRC. These lesions, if not treated, can evolve in the deeper muscular layers and throughout the intestinal wall, culminating in the development of CRC (26).

## The relationship between gut microbiota and colorectal carcinogenesis

Dysbacteriosis leads to the development of several diseases, especially those of an inflammatory order, such as IBD and CRC (52). The etiology of CRC can be attributed to the proliferation of *H. pylori, Streptococcus bovis* Orla-Jensen, *Enterococcus faecalis* Schleifer and Kilpper-Bälz (1984) and Orla-Jensen (1919), *Clostridium septicum, Escherichia coli* T. Escherich (1885)*, Fusobacterium* spp. Knorr (1922), *Bacteroides fragilis* (3, 8) and *Streptococcus gallolyticus* (3) (Table S1).

These microorganisms (Table S1) are etiologically related to CRC through innumerable effects. *H. pylori* have been associated with CRC, especially in hypergastrinemia relapses cases (8, 53). Already the relation of the bacterium s. bovis with CRC occurs due to the installation of a pro-inflammatory process that can lead to the recruitment of leukocytes and the development of pre-neoplastic lesions (54).

The carcinogenic property of *E. faecalis* occurs in bacteremia cases, characterized by frequent bacterial translocations with consequent increase in intestinal permeability, in addition to reactive oxygen species (ROS) formation (55). The intestinal permeability increase could also be a consequence of *E. coli* translocation to the intestinal region, causing serious epithelium lesions. Such deleterious effect may be related to toxins release and chronic inflammatory process development (8, 56). In such case *C. septicum* virulence and its ability to translocate to intestinal tissue may justify a strictly relation to CRC development (57). Fusobacterium and *B. fragilis*, from production and release of enterotoxins stimulate the immune response, causing alterations in intestinal epithelial cells (58, 59). These pre-neoplastic CRC effects caused by these microorganisms may occur in association. Besides that, Gagnière et al. (8) cite and establish an interconnection between genotoxins and bacterial metabolism, in addition to oxidative stress and inflammatory process, demonstrating the bacterial etiological multidirectionality of CRC.

## Exploring interactions among immune responses, intestinal microbiota and colorectal carcinogenesis

The gastrointestinal tract of the host is composed of thick layers of mucus. These layers are composed of mucin glycoproteins and other antibacterial factors, such as C-α-defensins lectins, lysozyme and phospholipase A2, which are excreted by Paneth cells. When in homeostasis, these substances confer perfect regulation on microbial abundance and the immune response. Otherwise, the development of intestinal dysbiosis is established (60).

**Figure 1:**
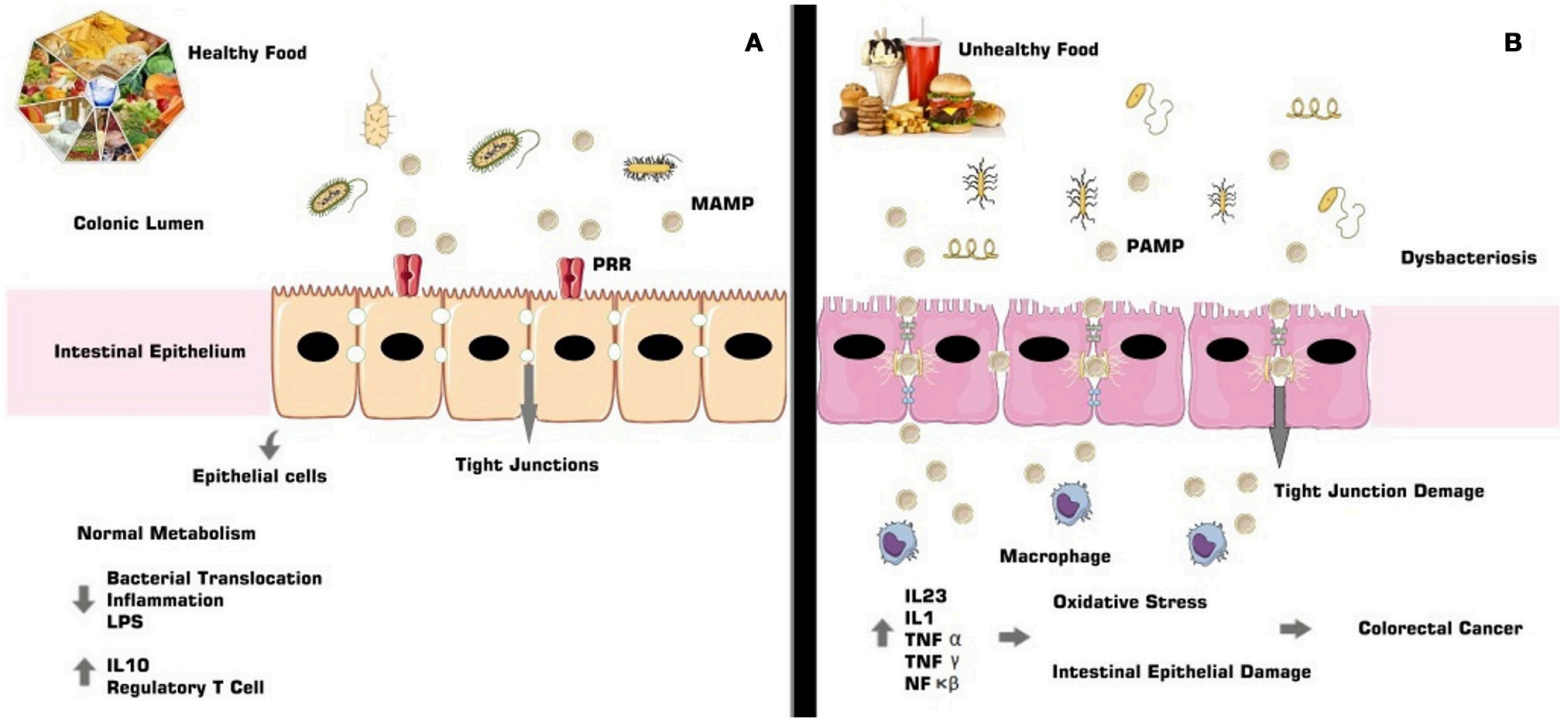
The gut microbiota composition is directly involved with immunologic and intestinal homeostasis **(A)**. In dysbacteriosis the signaling cascade results in the activity of specific receptors and their ligands, leading to the release of pro-inflammatory agents, such as cytokines and interleukins, allowing oxidative damage, associated with non-intestinal epithelial lesions, and consequent development of CRC **(B)**.

Complementing the intestinal immune defense, there are receptors whose function is to mediate the recognition process. These are the so-called PRRs that initiate the step of recognizing pathogen-associated molecular patterns (PAMPs). There are also toll-like receptors, of glycoprotein nature, known as the interface between the intestinal epithelial barrier and the immune system, which synthesize and release cytokines and chemokines and are responsible for the transcription of genes related to the triggering of the immunologic response. These receptors compose a singular superfamily called interleukin-1 receptors/toll-like receptors and are located in innumerable cells, in the intestinal epithelial barrier and in intracellular compartments (61, 62) (Figure 1).

Dysbacteriosis promotes the development of an inflammatory process characterized by increased expression of pro-inflammatory genes and specific receptors such as TLR3 and TLR4, activating NF-κB and IRF3 (interferon 3 regulating factor) (16, 61), besides the immunological agents MAPK (mitogen-activated protein kinase), AP1 (activator protein 1), STAT (signal transducer and activator of transcription), and IRF3 (interferon IFN-regulatory factor 3) (63).

Metabolic pathways, such as TLR4, are sensitized and activated by endotoxins found in Gram-negative bacteria, called lipopolysaccharides (LPS), which when released by bacterial lysis are mediated by acute inflammatory factors such as NF-κB (64). This inflammatory response to LPS occurs in the presence of cell and platelet adhesion molecules, pro-inflammatory cytokines [TNF-α, interleukin 6 (IL6), interleukin (IL1)], enzymes iNOS (nitric oxide synthase) and COX-2 (cyclooxygenase 2) receptors, bradykinin B1 receptors and the IkB-α repressor protein (63, 65).

The inflammatory process involved in dysbacteriosis can be the etiological cause of CRC and is associated with hyperplastic damage in the intestinal epithelium and with *lamina propria* fragmentation. These characteristics confer an environment that is conducive to bacterial translocation and to the development of pro-neoplastic lesions (3, 66, 67).

Inflammation of the intestinal tissue, due to a dysbacteriotic process, for example, is accompanied by the extinction of tumor suppressor genes and of genes that aid in the repair of genetic material. This also produces inflammatory-immunological conditions that will lead to the development of an environment conducive to the cancerous installation (68). In the inflammatory-immunological process that is characteristic of dysbacteriosis and that can cause CRC, activation of the NF-κB pathway occurs, and this then causes conformational changes in the neoplastic environment, stimulates action of the pro-inflammatory cytokines, such as IL6, which has an important pathogenic role in the progression of CRC and regulates genes related to the tumor necrosis factor (TNF) and COX-2 enzyme, both highly expressed in neoplastic conditions (66). Regulatory T cells are also expressed in inflammatory conditions that can lead to CRC (3).

Therefore, it is verified that the gut microbiota and its composition (specific microorganisms) are important promoters of the inflammatory-immunological response, conditioning or not the development of a pro-mutagenic environment for CRC (69, 70).

## Functional foods as gut microbiota modulators and chemopreventive action

The modulation of the intestinal microbiota and its consequent beneficial effects, which include protection against pathogenic invaders, immune system stimulation, and selective beneficial bacteria growth, occurs through the use of prebiotics, probiotics (71) and the association of the two in varying amounts forming the symbiotic (71–74).

In examining this association, it is important to consider the most common phyla identified in most individuals (Firmicutes and Bacteroides and, to a lesser extent, Actinobacteria and Proteobacteria) (4, 75) and the constant adaptation and quantitative and qualitative modification of the intestinal microbial composition. These modifications interfere in physiological, metabolic and immunological effects, considering the close relationship between the healthy process and disease (76).

Therefore, intestinal microbial community modifications that culminate in the extinction of the community's balance and the characterization of an intestinal dysbiosis status, as previously reviewed, can be related to the onset of chronic non-communicable diseases (NCDs), such as obesity, insulin resistance and cardiovascular diseases (77, 78), and particularly to CRC (3). Considering the benefits and effects of modulation of the gut microbiota, especially in the prevention and treatment of CRC, there are functional foods that motivate countless studies, with the objective of identifying their properties and applications. Functional foods are composed of bioactive ingredients with beneficial properties for metabolic and physiological well-being (79, 80).

Future prospects indicate that the constitution of the intestinal microbiota can be enhanced by the insertion of functional foods into the diet, with special emphasis on chemopreventive, biologically active compounds present in foods of plant origin and that can prevent and/or attenuate the development of CRC (81, 82).

In this case, flexseed (*Linum usitatissimum* L.) (83, 84), oat bran (*Avena sativa* L.) (85) and soybean *Glycine max* L.) (86), foods that are sources of PUFAs, especially the n-3 series (11), soluble food fibers (11, 12) and phytochemicals and antioxidants (87) (Table S2).

**Figure 2:**
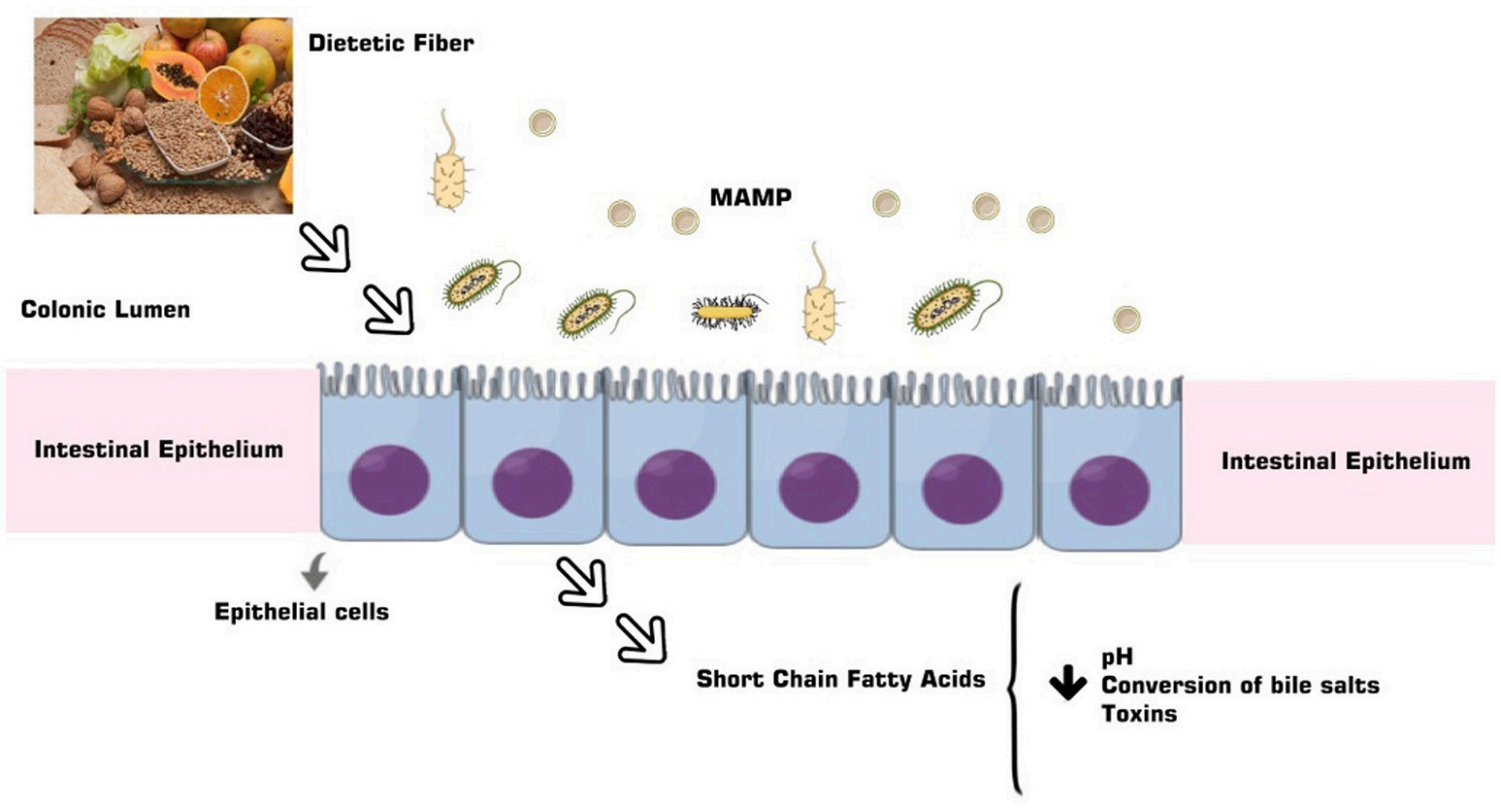
The PUFAs derive from a complex relationship between the gut microbiota and the diet, besides the characteristics of the microbiome, and are a product of the fermentation of dietary fiber and lignan absorbed by the small intestine.

**Figure 3:**
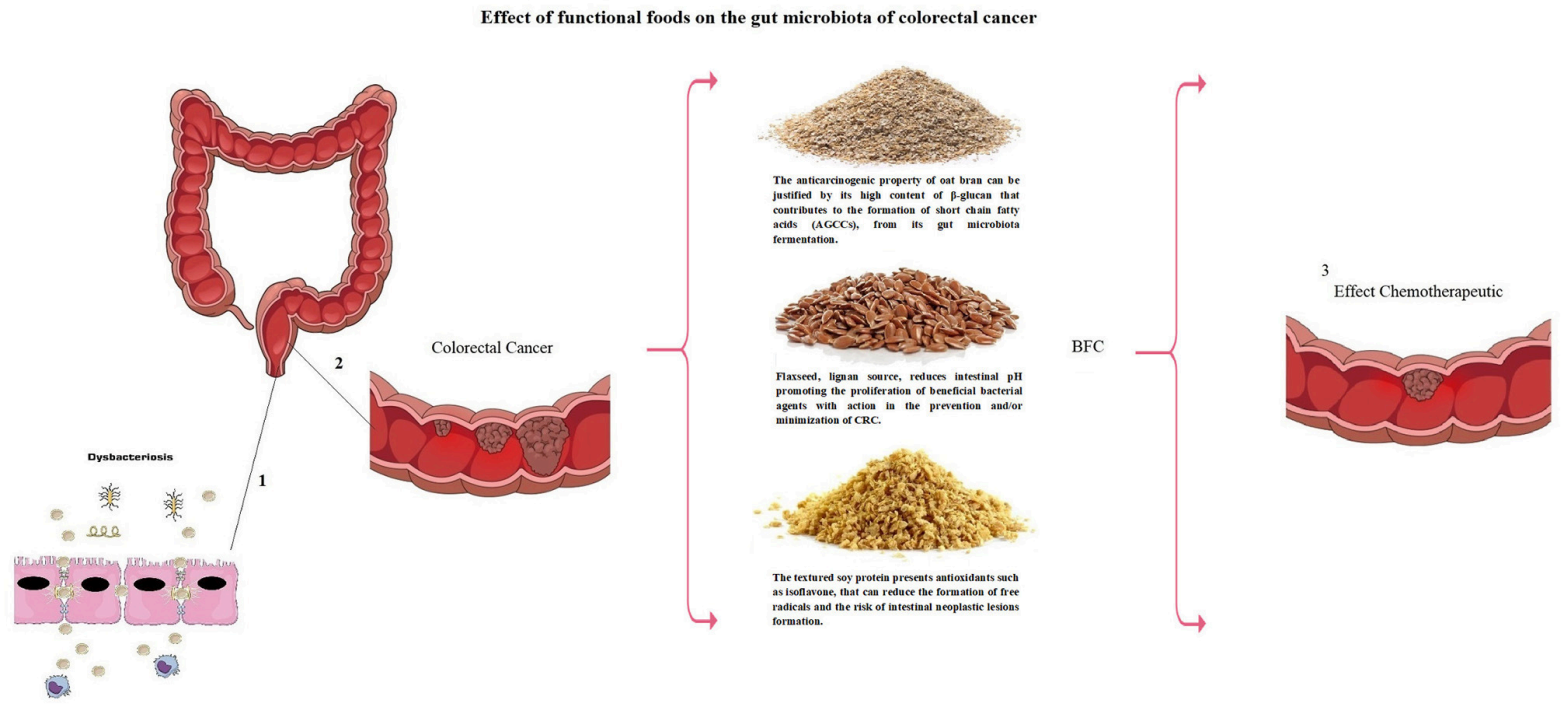
Dysbacteriosis process (1) causing colorectal cancer (2). Modulation of gut microbiota may be conducted by means of functional foods (BFC: Bioactive Food Compound—National Institute of Industrial Property – n. BR 10 2013 018002 5) with preventive and therapeutic action for colorectal cancer (3).

The anticarcinogenic property of soluble fibers like lignan and β-glucan could be justified by short chain fatty acids (SCFAs) formation, in special acetate, propionate and butyrate, from its gut microbiota fermentation (Figure 2). The SCFAs are responsible for the intracellular and colonic pH decrease, making the intestinal environment more acidic. Such condition inhibits pathogenic organism proliferation, toxic degradation products formation (88), DNA damage induction (89), the cell proliferation and increase apoptosis of tumor cells (90, 91). These factors could be reduced by dietary fibers that also have the capacity to act in the pre-neoplastic process, with a reduction in the inflammatory and oxidative process (73, 92) (Figures 1B, 3). However, such favorable CRC effects could be perceived in a dose-dependent manner (93). The ability to block neoplastic cell proliferation and induce apoptosis, as well as pre-neoplastic CRC conditions, is also a feature of n-3 series PUFAs. These lipids also could alter the cell cycle components and acts on the immune system markers and gene expression modulation by regulating CRC related genes expression (94, 95) (Figures 1B, 3). These effects according to D'Eliseo and Velotti (96) are perceived at different periods and exposure doses. Antioxidants, especially the isoflavones, genistein, and daidzein, block the DNA bases attack, avoiding the lesions formation and cellular integrity losses, due to free radicals' neutralization generated by cellular metabolism or exogenous factors (97, 98). In contrast, high food consumption when submitted to the fermentation process may be related to the increased risk of developing CRC (99). The other protective effects could be related to damaged cell membranes reconstitution and further DNA molecule damage removal, since oxidative stress may be directly related to the pre-neoplastic conditions in phases of initiation and promotion of cancer (100). Besides of the inflammatory process with increased activity of inflammatory cytokines and intestinal permeability (101) (Figures 1B, 3).

The immunomodulatory effects of flaxseed are perceived from its prebiotic properties. Because it is loaded with mucilage, this food maintains the integrity of the intestinal epithelial barrier, minimizing inflammatory processes, guaranteeing the proliferation of beneficial phyla to the detriment of putrefying and harmful species. This modification guarantees an improvement in the immune defense of the host, reducing the risk of developing NCDs, especially IBDs and CRC (102). The prebiotic effect of flaxseed is also presented by oats and soybeans. These two functional foods are filled with soluble fiber, contributing to the balance of the gut microbiota from its modulation. Gut microbiota modulation promotes the proliferation of probiotic phyla, guaranteeing the absorption of nutrients, protection against pathogens, and modulation of the immune system (103, 104).

The modulation of the gut microbiota composition from these functional foods also occurs in the presence of phytochemicals. Maintaining the balance of the gut microbiota ensures perfect immune defense and disease prevention, specifically CRC, decreasing cell proliferation, stimulating the induction of apoptosis, inhibition of angiogenesis and delay of the metastatic process (105, 106).

Finally, these functional foods (flaxseed, oat bran, and soybean) form a bioactive food compound (BFC) that is patented by National Institute of Industrial Property (number BR 10 2013 018002 5), published by Ministry of Development and Foreign Trade, Brazil in august 2015. BFC is rich in proteins, in PUFAs (60.41%) (n-3 series (30%) and n-6 series (27%)) and also in dietary fibers. Among such fiber it is possible to find soluble fibers including lignan and β-glucans, both with high viscosity and fermentable potential (107). Moreover BFC has been studied as adjuvant therapy in the NCDs directly related to metabolic syndrome (MS) including atherosclerosis. Such food composition exhibit low atherogenicity, thrombogenicity, and hypocholesterolemia/hypercholesterolemia, also acting at anthropometric measurements reduction (108, 109). Additionally studies with BFC demonstrate direct chemopreventive and/or chemotherapeutic relation with CRC (85, 86, 94, 110).

Therefore, a diet rich in vegetables, fruits, oilseeds, low glycemic complex carbohydrates, mono, and polyunsaturated fats and proteins presents a good balance in quality and quantity of phytochemicals, especially phenolic compounds such as flavonoids. These substances have considerable antioxidant activity, a property associated with a lower incidence of NCDs and lower resulting mortality, specifically CRC (111, 112).

The chemopreventive agents found in functional foods can be used with a powerful preventive effect, as their multiple mechanisms of action aid in the treatment and in the reduction of the risk of CRC recurrence, besides contributing to the improvement of the quality of life of the carriers of CRC in Figure 3.

However, it is noted with this review that there is a potential number of studies demonstrating the role of the intestinal microbiota and its imbalance at CRC pathogenesis. Associated with this problem are the functional foods that may act as anticarcinogenic and chemotherapeutic in animal models, being the model of choice for most research in this emerging field. This review proposes the continuity of potent studies that demonstrate causality in various experimental and human models, since some observations in animal models may not be applicable in people, although both present some significant similarities, genetic, anatomical, and physiological factors differ (113–115).

## Conclusion and future prospects

Dietary modulation of the intestinal microbiota, avoiding the intake of foods that stimulate dysbiosis and intestinal inflammation, is an efficient strategy to prevent the development of CRC. In this review, several studies have demonstrated the functional chemopreventive and/or chemotherapeutic value of the foods that compose the BFC in CRC. These effects may stimulate the use of flaxseed, oat bran and soya in the diet, as part of sweet and savory preparations, as well as in the industrial production of cereal bars and added to bakery products.

Future prospects point to the recommendation of a specific dietary plan, from the ingestion of functional foods that compose the BFC, highlighting the importance of its bioactive compounds, versatility of use and ease of access. The functional properties and the economic character of these functional foods demonstrate a participation in reducing health costs and improving the quality of life of the general population, especially in the prevention and treatment of NCDs, with an emphasis on CRC.

Taking advantage of the socio-demographic aspects and economic opportunities that favor the use of BFC in CRC, it is proposed that studies on this subject should continue, considering the need for clarification on the synergism and/or antagonism between bioactive compounds, as well as the daily dose of use and the possible mechanisms of action of these food substances.

It is concluded with this review that modifiable lifestyle factors such as western standard diet with high glycaemic load, unfavorable energy balance (obese or physically inactive) and smoking may have an important role in the formation of the intestinal microbiota, which is determinant for a favorable prognosis in CRC. Related to this, it is important to consider intraindividual differences in diet response as well as the great difficulty of nutrition and food professionals in stimulating compliance with these dietary recommendations.

## Author contributions

LM and RdSF drafted the manuscript, composed the figures, and critically revised the manuscript. LM, RdSF, and RdCAG conceived the manuscript and finalized the draft. RM have written some part. RdCAG, AdC, OF, RM, and CC revised the manuscript. All authors read and approved the final manuscript. RM wrote the bioactive compounds and that revised the final version of the manuscript.

### Conflict of interest statement

The authors declare that the research was conducted in the absence of any commercial or financial relationships that could be construed as a potential conflict of interest.
