# Supplementary material for: The Complex Puzzle of Interactions Among Functional Food, Gut Microbiota, and Colorectal Cancer
Source: Front Oncol. 2018 Sep 5;8:325. doi: 10.3389/fonc.2018.00325 (PMC6133950; doi:10.3389/fonc.2018.00325)
Supplement: Supplementary file 1 [file Table_1.docx]

Supplementary Material

**The Complex Puzzle of Interactions among Functional Food, Gut Microbiota and Colorectal Cancer**

Lígia Aurélio Bezerra Maranhão Mendonça^*1^, Rosângela dos Santos Ferreira ^2*^, Rita de Cássia Avellaneda Guimarães ^2^, Alinne Pereira de Castro^1^, Octávio Luiz Franco^1,3^, Rosemary Matias^4,5^, Cristiano Marcelo Espinola Carvalho^1,5^

*** Correspondence:** Lígia Aurélio Bezerra Maranhão Mendonça lmendoncanutri@gmail.com

# 1 Supplementary Table 1

**Table 1.** Studies associated with various *phyla* of microorganisms and their etiological link with the CRC

| **MO^*#^** | **Population** | **Identification Method** | **Conclusion of the Authors** | **Ref.** |
| --- | --- | --- | --- | --- |
| *H. pylori* | Patient with CRC *vs* Patient with colon polyps | Endoscopy, IgG serology, Hp, IgG and CagA expression and biopsy | Non-significant association | [55] |
| *S. bovis* | Patients with bacteremia of *S. bovis* | Colonoscopy | - Approximately 30.6% of the patients studied presented CRC | [54] |
|  | Patients randomly selected (n= 203) |  | 24 % of the patients had bacteremia of *S. bovis*, of these approximately 35 % presented CRC | [55] |
| *S. gallolyticus* | Patients with CRC *vs* colonic mass of patients “healthy” | qPCR | Present but low prevalence of *S. gallolyticus* infection | [56] |
| *E. faecalis* | Patients with bacteremia of *E. faecalis* | Colonoscopy | Infiltration of CRC (bacterial translocation) | [57] |
| *C. septicum* | Patient with necrotizing fasciitis | Colonoscopy and histological methods | High virulence of the *C. septicum* is related to the development of CRC | [58] |
| *E.* *coli* | Patients with CRC *vs* Patients with uncomplicated diverticulitis | Biopsy of the intestinal epithelium and pathological methods | Relationship among unfavorable prognosis and colonization of colonic mucosa by *E. coli* in both conditions | [59] |
|  | Patients with CRC | Biopsy of the colonic mucosa and standard biochemical methods | High incidence of *E. coli* in patients with CRC | [60] |
|  | Patients with CRC  (n= 55) | qPCR | Important association between bacterium *E. coli* and CRC | [61] |
| *Fusobacterium* | Patients with CRC | PCR, MSI, DNA methylation, KRAS, BRAF, mutations of PIK3CA | The increase of phylum is reflected pathogically in the intestinal microbiota, immune control and neoplasia | [62] |
|  | Patients with CRC  (n= 55) | qPCR | Important association between the *phylum* and CRC | [61] |
| *B. fragilis* | Patients with CRC  (n= 155) | Colonoscopy | Positive association between the present bacterium and CRC | [63] |
|  | Patients referred for colonoscopy (n= 150) | qPCR | Important association between the bacterium *B. fragilis* and CRC | [63] |

*MO: Micro-organisms. #Natural Reservoir: gastrointestinal tract.
