# Supplementary material for: The Complex Puzzle of Interactions Among Functional Food, Gut Microbiota, and Colorectal Cancer
Source: Front Oncol. 2018 Sep 5;8:325. doi: 10.3389/fonc.2018.00325 (PMC6133950; doi:10.3389/fonc.2018.00325)
Supplement: Supplementary file 2 [file Table_2.docx]

Supplementary Material

**The Complex Puzzle of Interactions among Functional Food, Gut Microbiota and Colorectal Cancer**

Lígia Aurélio Bezerra Maranhão Mendonça^*1^, Rosângela dos Santos Ferreira ^2*^, Rita de Cássia Avellaneda Guimarães ^2^, Alinne Pereira de Castro^1^, Octávio Luiz Franco^1,3^, Rosemary Matias^4,5^, Cristiano Marcelo Espinola Carvalho^1,5^

*** Correspondence:** Lígia Aurélio Bezerra Maranhão Mendonça lmendoncanutri@gmail.com

# 1 Supplementary Table 2

**Table 2.** Functional foods, BFC constituents, used in the treatment of CRC

| **Biological assay** | | | | |
| --- | --- | --- | --- | --- |
| - **Functional Food** | - **Bioactive Substance** | **Probable bioactive effect** | **Population** | - **Ref.** |
| Flaxseed | - Polyunsaturated fatty acid (n-3) | Inhibition of angiogenesis caused by CRC | Nude (nu/nu) | - [95] |
|  |  | - Increased expression of FFAR4 in colon | Sprague–Dawley rats | - [96] |
|  | Phytic Acid  Inositol Hexaphosphate (IP6) | Inhibition of development of metastatic progression of CRC | BALB/c  (n = 48) | - [97] |
|  | Lignan | Decreased proliferation and increased apoptosis of tumor cells - CRC | - Caco-2 colon cancer cells | - [98] |
| Oat | *β* glucan | Prevention of CRC | - Kunming Mice | - [92] |
|  | Phenolic amides (Avenanthramides) | Reducing the risk of CRC | Caco-2 and HT29 colon cancer cells | - [99] |
|  |  | Induction of apoptosis of colon cells | HCT-116 human colon cancer cells | [7] |
| Soy | Isoflavone (Genistein and daidzein) | Suppression of colon cancer growth | Sprague–Dawley rats | - [100] |
|  |  | - Reducing the risk of CRC - Anti-cancer | Women  (n = 68.412) | - [101] |
|  |  |  | - Patients with CRC (n = 901) | - [102] |
|  |  |  | - Patients with CRC (n = 101) | - [93] |
| **Reviews and Other Studies** | | | | |
| - **Functional Food** | - **Bioactive Substance** | **Probable bioactive effect** | | - **Ref.** |
| Flaxseed | - Polyunsaturated fatty acid (n-3) | Blocks tumor formation | | - [103] |
|  |  | Deregulates the expression of genes involved in the CRC and alters the membrane lipid composition of tumor cells.  Reduction of proliferation and induction of apoptosis. | | - [104] |
|  | IP6 | Inhibition of the metastatic process in CRC | | - [96] |
|  | Lignanas   - (Secossolariciresinol diglucoside) | Reduction of proliferation and induction of tumor cell apoptosis | | - [103] |
|  |  | Modulates cellular signaling pathways | | - [105] |
|  |  | Antiproliferative and anti-angiogenesis protection | | - [106] |
|  | Phenolic amides (Avenanthramides) | Reducing the risk of CRC | | - [107] |
| Oat | *β* Glucan | Formation of short chain fatty acids (SCFA) that decrease intra-colonic pH, inhibiting pathogenic and toxic proliferation (Figure 2) | | - [108] |
| Soy | Isoflavone (Genistein and daidzein) | Inhibit cell proliferation and induce apoptosis; Decrease GLI1 expression | | - [109] [110] |
|  |  | Inhibition of MMP-9 (Matrix metallopeptidase 9) responsible for tumor progression | | - [111] |
|  | Saponins | Inhibition of MMP-9 and gelatinase B, responsible for tumor progression | | - [111] |
